# Increased Risk for Clinically Significant Sleep Disturbances in Mild Traumatic Brain Injury: An Approach to Leveraging the Federal Interagency Traumatic Brain Injury Research Database

**DOI:** 10.3390/brainsci14090921

**Published:** 2024-09-14

**Authors:** Maya E. O’Neil, Danielle Krushnic, William C. Walker, David Cameron, William Baker-Robinson, Sara Hannon, Kate Clauss, Tamara P. Cheney, Lawrence J. Cook, Meike Niederhausen, Josh Kaplan, Miranda Pappas, Aaron M. Martin

**Affiliations:** 1 HSR Center to Improve Veteran Involvement in Care (CIVIC), Portland VA Health Care System, Portland, OR 97239, USAsara.hannon@va.gov (S.H.);; 2 Department of Clinical Psychiatry, Oregon Health & Science University, Portland, OR 97239, USA; 3 Department of Medical Informatics and Clinical Epidemiology, Oregon Health & Science University, Portland, OR 97239, USA; 4 Department of Physical Medicine and Rehabilitation (PM&R), School of Medicine, Virginia Commonwealth University, Richmond, VA 23298, USA; william.walker@vcuhealth.org; 5 Richmond Veterans Affairs (VA) Medical Center, Central Virginia VA Health Care System, Richmond, VA 23249, USA; 6 Department of Pediatrics, School of Medicine, The University of Utah, Salt Lake City, UT 84112, USA; 7 OHSU-PSU School of Public Health, Oregon Health & Science University, Portland, OR 97239, USA; 8 Department of Neurology, Oregon Health & Science University, Portland, OR 97239, USA; 9 Pacific Northwest Evidence-Based Practice Center, Oregon Health & Science University, Portland, OR 97239, USA; 10 Mental Health and Behavioral Science Service, James A. Haley Veterans Hospital, Tampa, FL 33612, USA; 11 Department of Psychiatry and Behavioral Neurosciences, University of South Florida, Tampa, FL 33612, USA

**Keywords:** traumatic brain injury, sleep disturbance, insomnia, evidence synthesis, data repository, meta-data

## Abstract

Study Objectives: The Federal Interagency Traumatic Brain Injury Research (FITBIR) Informatics System contains individual-patient-level traumatic brain injury (TBI) data, which when combined, allows for the examination of rates and outcomes for key subpopulations at risk for developing sleep disturbance. Methods: This proof-of-concept study creates a model system for harmonizing data (i.e., combining and standardizing data) across FITBIR studies for participants with and without a history of TBI to estimate rates of sleep disturbance and identify risk factors. Results: Three studies were eligible for harmonization (N = 1753). Sleep disturbance was common among those with a history of mild TBI (63%). Individuals with mild TBI were two to four times more likely to have sleep disturbance compared to those with no history of TBI. Conclusions: This study established methods, harmonization code, and meta-databases that are publicly available on the FITBIR website. We demonstrated how the harmonization of FITBIR studies can answer TBI research questions, showing that associations between TBI and sleep disturbance may be influenced by demographic factors.

## 1. Introduction

Sleep disturbance is highly prevalent following traumatic brain injury (TBI) [1,2], persists across the recovery trajectory, and can reflect several clinically significant disorders. For instance, as many as two-thirds of individuals hospitalized post-TBI have previously undiagnosed sleep apnea upon criterion standard screening during inpatient neurorehabilitation [3]. Insomnia disorder is estimated to occur in more than 25% [4] of individuals following TBI. Epidemiologic work demonstrates that sleep disorders are some of the most prevalent conditions at 2- and 5-years post-TBI [5,6], and as many as one-fifth of Veterans develop any sleep disorder within the first 5 years following TBI [7].

Sleep health is critical in the process of neural recovery from TBI [8], with glymphatic clearance of interstitial waste becoming disrupted in the presence of unaddressed sleep disturbance [9]. There is a reciprocal relationship with injury-induced neuroinflammatory processes contributing to disrupted sleep regulation, and poor sleep in turn promoting neuroinflammation [10]. Sleep disturbance following TBI has been shown to be associated with many recovery-based outcomes [11] including reduced cognitive improvement [12,13,14], lengthier posttraumatic amnesia [15], delayed participation and engagement in rehabilitation therapies and worse outcomes [16,17], higher risk for affective disorders [18,19], poorer trauma recovery [20,21], increased pain intensity [11], worse headache severity [22], and poorer quality of life [23,24].

The risk of sleep disturbance following TBI may vary across populations and levels of TBI severity [25] and is influenced by several factors including age [26], mechanism of injury [27], presence of posttraumatic stress disorder (PTSD) [28], symptom severity [20], and type of sleep disturbance. A Taiwanese study of 80 matched controls of older individuals (>65 years) who received treatment for first-time TBI found that being male was the only significant risk factor for obstructive sleep apnea, while increased pain and depressive symptom severity was predictive of post-TBI insomnia [24]. A larger study of over 98,000 US Veterans with TBI and age-matched peers without TBI found that, after adjusting for relevant covariates (e.g., demographics, medical and psychiatric history), those with TBI were 41% more likely to develop any sleep disorder over a 14-year period post-TBI [7]. This association was found to be stronger for mild TBI and similar for those with and without PTSD diagnoses.

### 1.1. Addressing Methodological Limitations in the Extant Literature

Clarity on the natural history and risk prediction of sleep disturbance outcomes associated with TBI has been hampered by the lack of large, longitudinal datasets with detailed information on multiple patient groups. Heterogeneity of populations, injury characteristics, and outcome measurements have created further challenges. Existing data may be leveraged to address these concerns particularly through FAIR (Findable, Accessible, Interoperable, and Reusable) data practices, which encourage the reuse and sharing of data to advance scientific discovery [29]. In keeping with these recommendations, the National Institutes of Health (NIH) and the US Army Medical Research and Development Command (USAMRDC) collaboratively funded and developed a biomedical informatics system and data repository for TBI research, the Federal Interagency Traumatic Brain Injury Research Informatics System (FITBIR; https://fitbir.nih.gov/) (accessed on 21 September 2021), to better harness the power of large-scale data across multiple studies. FITBIR was developed as a publicly available data platform of TBI-focused human-subjects research studies, enabling users to access deidentified study data for TBI research.

FITBIR is unique in that it contains individual-patient-level data, which when combined, allows for the examination of rates and outcomes for key subpopulations (e.g., gender, historically marginalized/underrepresented groups, members of the military/Veterans) that may be underrepresented in individual studies. This approach is akin to gold-standard individual-participant-level reviews, which are relative to reviews that use study-level data, avoid bias, allow for the appropriate management of missing data, and result in more robust and nuanced analyses [30]. Thus, analyzing such pooled data would inform early recognition and treatment of sleep disorders as a risk factor for the development or exacerbation of other conditions. The ability to stratify risk around sleep disturbance and ultimately sleep disorders is likely to aid in increasing access to and engagement in evidence-based, guideline-concordant first-line treatments for a population that experiences challenges with this care [31,32,33,34].

A lack of data access at the participant level and lack of data harmonization (i.e., the process of combining and standardizing data from multiple sources) are current barriers to the analysis of pooled datasets for understanding sleep and other TBI-related outcomes. The FITBIR database was developed to overcome the data access limitation, with a steadily increasing number of TBI studies submitting data to this platform. However, because studies use divergent measures for various constructs including TBI characteristics, demographic factors, and comorbidities, data harmonization continues to be a formidable barrier for these types of analyses. For example, some studies may have dedicated sleep disturbance measures, while others may be reflected in items embedded in measures assessing other constructs (e.g., a sleep question item on the Patient Health Questionnaire (PHQ-9) for measuring depression) [35]. At the time of this study, data are still being added to FITBIR; however, in order to make this dataset more usable and widely disseminated, it is necessary to establish data harmonization and analysis procedures to pool study and individual-participant-level data into a single meta-dataset. Therefore, our overarching goal was to utilize the publicly available data in FITBIR to develop a model system which can identify common measures and pool data across TBI studies. Our efforts were initially focused on psychological variables commonly occurring with TBI including sleep disturbance, which is the focus of this subproject.

### 1.2. Objectives

Though relatively few studies were publicly available in the first few years that FITBIR was established, and datasets were being cleaned and contributed, more are becoming publicly available for follow-up analyses over time. Therefore, this study describes our preliminary steps to leverage FITBIR to examine individual-participant-level data across TBI studies contained within that publicly available database for combination and analysis. Our overarching project, “FITBIR: Accelerating Synthesis of TBI Research Using Novel Methods (FASTRUN)”, harmonized data related to mental health, quality of life, and cognitive functioning from shared, publicly available studies in the FITBIR database. The specific aims of this sleep disturbance subproject are as follows:

Aim 1: Develop a model system for harmonizing data for key variables across FITBIR study datasets resulting in an integrated database containing data for participants with and without a history of TBI.
Aim 2: Use merged datasets to estimate rates of sleep disturbance and identify outcome risk factors.
Aim 3: Develop and share the methodologic products (e.g., the code and quantitative analysis syntax) created for this research project that can be used to facilitate more rapid synthesis of FITBIR data in the future as more studies are added to the publicly available database.

## 2. Methods

Our overarching project harmonized data from shared, publicly available studies in the FITBIR database to create common measures and variables allowing data to be combined across studies, with an emphasis on psychological health outcomes. In addition to measures of sleep disturbance, these included PTSD, depression, suicidal ideation, alcohol use disorder, cognitive/neurologic disorders, and substance use disorder, as well as functional health (e.g., quality of life, employment status, and functional ability).

This secondary data analysis subproject focused on identifying, harmonizing, and analyzing studies reporting on sleep disturbance in the shared datasets. We hypothesized that outcomes from publicly available studies in FITBIR would support the existing literature, which suggests that history of mild TBI is associated with higher rates of sleep dysfunction compared to no TBI history. We further hypothesized these associations would differ by patient and injury characteristics. We conducted exploratory analyses to examine associations between TBI, sleep disturbance-related outcomes, and other demographic characteristics (e.g., gender, race/ethnicity, age, and level of education).

Data harmonization is the process of combining and standardizing data from multiple sources, in our case, data from studies in the FITBIR data repository, into one unified dataset. In order to accomplish this, a FITBIR account was created, providing access to study data shared with the FITBIR data repository. Study data is available to view and download from the FITBIR website as .csv files with a FITBIR account. The unified dataset includes data from all possible studies where similar variables and measures were collected (e.g., demographics, TBI severity, depression measures, and suicidal ideation measures). Cross-sectional datasets were created by harmonizing data across studies with similar outcomes and merging key variables including TBI severity, demographic information, and sleep outcomes using RStudio version 3.6.1. The harmonized, cross-sectional datasets, methods, R syntax, and interactive data visualizations are shared on the FITBIR website so that future researchers, policymakers, and other stakeholders can access merged results for core FITBIR variables (https://fitbir.nih.gov/) (accessed on 21 September 2021). The meta-data and code from this study are available at https://fitbir.nih.gov/ (accessed on 21 September 2021) by navigating to the Meta Study tab, selecting study “FITBIR: Accelerating Synthesis of TBI Research Using Novel Methods”, and clicking on the Data tab. The meta-data and code are only available to those who have created a FITBIR account. The interactive data visualizations were created using R Shiny Apps and are available to the public on the FITBIR website, https://fitbir.nih.gov/meta_study_profile/223 (accessed on 21 September 2021), by navigating to the Data, then Submitted Data, and finally Meta-Studies tabs where the meta-datasets for this secondary data analysis are shared under the lead author’s name. The visualizations include a prevalence tab, providing a visual summary of the outcomes, TBI severity, and demographic information, and an effect size tab, which displays associations between mild TBI and outcomes using logistic mixed-effect regression models [36].

### 2.1. Study Approval

This research was reviewed and approved by the USAMRMC Office of Research Protections, Human Research Protection Office (HRPO), prior to implementation. We complied with FITBIR, Congressionally Directed Medical Research Programs, and local institutional review board (IRB) standards and requirements at Oregon Health and Science University (IRB number STUDY00021337) and the VA Portland Health Care System (IRB number 4621), including using approved methods of maintaining data behind secure firewalls and the transfer of data within and across sites. While use of deidentified data/records may be exempt from IRB review, we ensured that exemption was determined by the office of the institution’s IRB of record and confirmed by USAMRMC HRPO.

### 2.2. Study Inclusion/Exclusion Criteria

**Figure 1:**
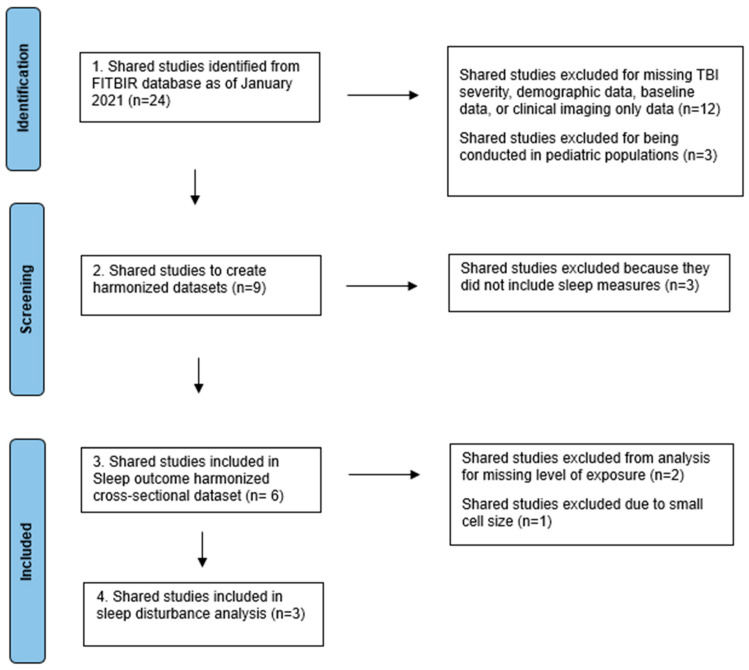
Flowchart.

At the initiation of this project in 2021, there were a total of 24 shared studies available in the FITBIR database [37,38,39,40,41,42,43,44,45,46,47,48,49,50,51,52,53,54,55,56,57,58,59] which were reviewed for relevance and inclusion, akin to an individual-participant-level data systematic review limited to studies contained in the FITBIR database [60,61,62,63]. To be included, studies had to report the severity of TBI (mild, moderate/severe, no TBI) and demographic information, include adult participants (≥18 years), measure at least one of the psychological or functional health outcomes relevant to our overall FASTRUN project (e.g., psychological health and functioning outcome data), and include an associated follow-up timepoint. Twelve studies were excluded for missing TBI severity, demographic information, baseline data, or relevant outcome data. An additional three studies were excluded because they did not include adult participants, leaving nine shared studies [40,42,43,45,46,47,48,54,57] to create a new, harmonized dataset (Figure 1). Of these, six studies [42,43,45,47,48,57] included sleep disturbance outcomes (Table 1) and were included in the cross-sectional dataset.

Due to varied information available in each study (e.g., different methods/measures used to characterize TBI), TBI severity was determined on a study-by-study basis. TBI severity for some studies was based on the inclusion criteria for the study (e.g., a study only included participants with mild TBI), while other studies included TBI severity variables to differentiate between participants with no TBI, mTBI, and moderate/severe TBI. Study 246 [42] used the GCS to determine TBI severity. GCS scores of 13–15 were classified as mild TBI, and GCS scores of 3–12 were classified as moderate/severe TBI. All participants had mild or moderate/severe TBI. Study 248 [43] included only participants with moderate/severe TBI. Study 254 [45] included participants with no TBI or mTBI. TBI severity was determined by the International Classification of Diseases (ICD)—Post-Concussive Syndrome (PCS) diagnostic criteria (ICDPCS). Studies 263, 264, and 326 [47,48,57] included participants with mTBI or no TBI, labeled as either cases or controls, respectively. We used this information to create a harmonized TBI-severity variable with three levels: no TBI, mTBI, and moderate/severe TBI. The third group, moderate/severe TBI, combined data for any participant with a history of either moderate or severe TBI. All summary and analysis data included all three TBI severity levels.

### 2.3. Measures

All sleep disturbance measures and individual sleep variables were dichotomized in cross-sectional datasets used for analysis and summary tables (Table 1). For studies that used more than one sleep measure or variable, a participant was labeled as positive if any (i.e., one or more) measure or variable indicated the participant had any type of sleep disturbance. Several validated instruments and cutoff scores were used in the studies to measure sleep disturbance. Many of these measures were used to assess other aspects of health and functioning (e.g., PTSD or depression symptoms), but because they included items related to sleep, we were able to include these relevant data. For such measures, sleep disturbance was classified using cutoffs related to those individual sleep-related items. Measures/items and corresponding cutoff scores included the following: an overall score of 10 or greater on the Insomnia Severity Index (ISI) [64,65,66], a score of 1 or higher on item 3 of the Patient Health Questionnaire-8 Item (PHQ-8) [67,68,69], a score of 2 or higher on item 20 of the Posttraumatic Stress Disorder Checklist Civilian Version (PCL-C) [70,71], an overall score of 8 or greater on the Pittsburgh Sleep Quality Index (PSQI) [72,73,74], a score of 1 or greater on the sleep-related item from the Sport Concussion Assessment Tool 3 (SCAT3) [75], a score of 3 or greater on item 5 of the Rivermead Post-Concussion Symptoms Questionnaire (RPQ) [76], or a score of 1 on the sleep-related item from the TRACK-TBI Neurologic Assessment [42] (see Table 1 for the clinical cutoffs applied). We used baseline measurements to create the dichotomous sleep-disturbance outcome variable, and baseline was defined as the earliest timepoint with sleep measurements.

For the cross-sectional analysis, we dichotomized race and ethnicity to categorize participants as either “Non-Hispanic White” or “Other” due to the differences in ways race and ethnicity were reported across the studies that prevented us from retaining more specificity. We used baseline age to create the continuous age variable, which was defined as the youngest age recorded for each participant. Gender was recorded on multiple measures for each shared study. The final gender variable used for summary tables and analysis was from the measure with the smallest amount of missing data in each study. Due to limitations of the existing data, gender was coded as binary (male or female).

### 2.4. Data Analysis

We based our data harmonization and analysis approach on methods used in individual-participant-level data meta-analysis methods to appropriately account for multiple-study data combination [77,78]. Creating the dataset involved combining data from numerous studies, forms, and variables in the FITBIR data repository, which was managed by creating a function using RStudio syntax that would extract the variables of interest as well as the timepoint for that measurement. The function produced a dataset in long format with each row containing the study ID, participant ID, value and timepoint of the measurement, and the name of the form, section, and variable. From this intermediate dataset, we filtered baseline values and dichotomized the outcome to create a cross-sectional dataset with one row per participant. Separately, we created a demographic dataset and a primary dataset, with one row per participant, that could be joined with any dataset of interest. The primary dataset includes information on TBI injury severity, date of concussion, time between TBI and the beginning of the study, and study-level data. The datasets were then joined with the sleep disturbance cross-sectional dataset for analysis.

We used multivariable logistic regression models to measure the association of sleep disturbance and a history of mild TBI (yes/no). Studies were included in the analysis if they had both levels of exposure (i.e., participants with no history of TBI and participants with a history of mild TBI). We built a separate model for each study with age, gender, and race included as covariates and a model with combined results, with study ID included as an additional covariate. Missing data was excluded with listwise deletion. Analysis was performed using SAS version 9.4.

Due to the limited overlap of harmonized variables across studies, there is a potential bias of unmeasured confounding in the analysis. Therefore, we calculated E-values to assess the potential contribution of unmeasured confounding on our results. The E-value is defined as the minimum strength of association that an unmeasured confounder would need to have with both the exposure and outcome, conditional on the measured covariates, to fully explain away a specific exposure–outcome association [79]. We used the E-value formula for odds ratios for common outcomes as the prevalence of sleep disturbance was higher than 15% in our sample [79].

## 3. Results

Overall, 3314 participants (range: 80 to 1539) from six studies [42,43,45,47,48,57] had data related to the presence/absence of sleep disturbance. Among the participants from the six studies, 58% (n = 1920) had a history of mild TBI, 27% (n = 903) had a history of moderate or severe TBI, and 15% (n = 491) had no history of TBI (Table 2, Figure S1 in the Supplemental Material). The participants were majority male (81%), identified as Non-Hispanic White (63%), and were military Veterans or active-duty servicemembers (55%); 42% were between 25 and 39 years old. Overall, 55% were categorized as positive for sleep disturbance.

We then measured the association between sleep disturbance and history of mild TBI (versus no history of TBI) among studies that included these comparators. Three of the studies with sleep disturbance data included in the aforementioned descriptive, cross-sectional analyses were excluded due to lack of TBI-negative comparators [42,43] or small cell sizes [45]. Overall, 1753 participants from three studies [47,48,57] were included in this analysis (Table 3). The majority of participants were male (87%), between 25 and 29 years old (53%), and identified as Non-Hispanic White (61%); all participants from these studies were military Veterans or active-duty servicemembers. The prevalence of probable sleep disturbance ranged from 26% to 61%, and approximately 76% of participants from the three studies included in the comparative analysis had a positive history of mild TBI.

When data from each study was analyzed separately, participants with a history of mild TBI was associated with increased odds of screening positive for a sleep disturbance in all three studies compared to participants with no history of TBI (Table 4). One study was not included in the combined (pooled data) results for TBI-positive and TBI-negative comparators because it did not report race, but was analyzed by itself [57]. In that study [57], participants with a history of mild TBI had 2.72 times the odds of having a sleep disturbance compared to those without a history of TBI (95% CI: 1.06, 6.98).

**Figure 2:**
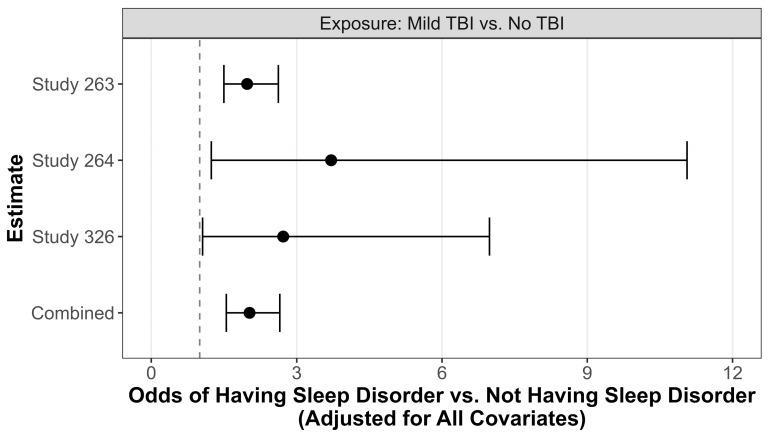
Effect size plot—odds of having sleep disturbance.

In the combined results of the other two studies, participants with a history of mild TBI had 2.03 times the odds of having a sleep disturbance than those without a history of TBI (95% CI: 1.55, 2.65; see bottom of Table 4 and Figure 2). After adjusting for the effects of TBI in the multivariable model, participants identifying as Non-Hispanic White had 0.52 times the odds of having a sleep disturbance compared to those categorized as Other (95% CI: 0.42, 0.65). Participants who identified as male had 0.77 times the odds of having a sleep disorder compared to those who identified as female (95% CI: 0.55, 1.07), after adjusting for the effects of TBI, though this finding was not statistically significant. For every one-year increase in age, the adjusted odds of having a sleep disturbance decreased by 1.20% (OR = 0.988, 95% CI: 0.977, 0.999). The study the participant was enrolled in (263 vs. 264) was not associated with the presence of sleep disturbance (adjusted OR = 0.99, 95% CI: 0.61, 1.61).

We conducted a sensitivity analysis on the association between history of mild TBI and probable sleep disturbance by calculating an E-value. For the observed odds ratio of 2.03, the E-value was 2.20. Therefore, it would take a strong unmeasured confounder that was associated with both a history of mild TBI and probable sleep disturbance by an odds ratio ≥ 2.20 each to account for the observed association in the current study. For the lower confidence interval limit of 1.55, the E-value was 1.80. Therefore, it would take a moderately strong unmeasured confounder that was associated with a history of mild TBI and probable sleep disturbance by an odds ratio ≥ 1.80 each for the confidence interval to cross the null value of 1.

## 4. Discussion

This is the first published study to provide a methodology for harmonizing sleep disturbance measures for patients with TBI across studies within FITBIR and to demonstrate its application in understanding the prevalence and risk factors for experiencing sleep disturbance. Consistent with the prior literature, a high degree of sleep disturbance exists among individuals with TBI, with 59% of individuals with a history of TBI in our cross-sectional sample likely experiencing clinically significant sleep disturbance. As hypothesized, those experiencing mild TBI compared to individuals with no TBI were more likely to endorse symptoms consistent with sleep disturbance across self-report measures. The overall odds ratio of 2.03 in our combined analysis is higher than the hazard ratio of 1.41 reported by Leng and colleagues [7] for developing a sleep disorder following TBI. The Leng study [7] used a large scale VHA patient database that relied on ICD/chart diagnoses, so potential explanations for their lower odds ratio include more stringent diagnostic criteria being applied than the identification of general sleep disturbance and underrepresentation of sleep disorders that have historically gone undiagnosed. Robust prospective data are needed to better understand the trajectory of TBI and the associated sleep disturbance over time; however, if sleep disturbance fluctuates with TBI severity, it may be used as a prognostic indicator of recovery from the lingering functional impairments associated with TBI. Furthermore, given the greater odds of sleep difficulties among individuals with TBI, additional assessment and intervention efforts may strengthen existing TBI-related care protocols including neurotrauma services and rehabilitation efforts.

Race, age, and gender were additionally examined as potential factors associated with sleep disturbance risk. After adjusting for the effects of TBI, White, Non-Hispanic individuals had a reduced risk (OR = 0.52) of sleep disturbance compared to individuals identifying as Non-White or Hispanic. Although disparities in sleep duration and quality across race and ethnicity have been demonstrated in the broader sleep literature in the general population, considerable variability exists in the methodology and racial/ethnic groups included [80]. Among studies examining sleep disturbance in TBI, comparator groups are often matched [81] on race, or race is controlled for in analyses. Despite this, in TBI samples, there is some prior evidence that those classified as poor sleepers are more likely to be Non-White [82]. While age-related differences were seen in the present study, this finding was in the unanticipated direction, with increased age being protective against sleep disturbance. This association is relatively weak however, and may reflect a ceiling effect in the study sample, as there were a limited number of individuals across studies over the age of 65 and a relatively small number of individuals over the age of 50 represented. Additionally, a larger scale study by Albrecht and Wickwire [26] highlights that TBI is a risk factor for insomnia and any sleep disorder in those over 65 years of age, a population at particularly high risk of TBI. While gender differences were not seen, there was a trend for male participants to be at less risk for sleep disturbance. This trend is difficult to interpret given the relatively greater proportion of males (87%) across studies may have impacted the results. While measures were concerned with self-report sleep disturbance, which generally reflects insomnia, Wei and colleagues [24] found gender to be a risk factor for obstructive sleep apnea in older adults following TBI. Historically, their finding is consistent with the prevalence of OSA being greater among men and insomnia being more prevalent among women [83].

### Strengths/Limitations

This proof-of-concept project harmonizing and analyzing FITBIR data on TBI and sleep disturbance had both significant strengths and weaknesses common to other early-stage methods development projects. The creation of new data repositories takes extensive time and effort, and though FITBIR has been established for numerous years, the understandably slow buildup of publicly accessible data has meant that robust results from multiple FITBIR studies with similar outcomes is still a few years away. Additionally, pooled analyses of individual-participant-level data across multiple studies can result in more robust findings in spite of the significant time, effort, and expertise required to harmonize data prior to combined analysis [60]. Our project established the novel methods necessary for data and variable identification, harmonization, and analysis using FITBIR studies. A main goal was to advance FAIR (Findable, Accessible, Interoperable, and Reusable) data methods for TBI and sleep using the FITBIR data platform, and to that end, we shared our harmonization and analysis methods and code along with the meta-dataset on the FITBIR website. We also have ongoing funding through the Department of Defense to continue this work, updating our datasets and analyses with the newly available studies in FITBIR that have been added since 2021. Through sharing our code and meta-data, our hope is that other research teams will be able to use these resources and be further encouraged to contribute and publicly share their own primary data as well as meta-data from FITBIR-based evidence synthesis projects.

A main strength of this study was the use of the FITBIR database, which allowed us to integrate individual-participant-level data from several studies, resulting in a larger sample of individuals with TBI and sleep disturbance data. This is a more robust method than combining study-level summary data (e.g., averages) as is standard in meta-analyses [60]. However, there are several limitations worth noting. Many of the studies in FITBIR did not have the requisite sleep disturbance data to be included in the present project. Of the 24 shared studies, only nine had adequate demographic and TBI severity data to be harmonized, only six contained sleep disturbance data, and only three of those included comparator data for analysis; none of the remaining three studies contained a TBI cohort of severity greater than mild. This limited our analysis on the risk of sleep disturbance for individuals with mild TBI versus those without. Similarly, we were unable to examine differences in severity of sleep disturbance by TBI severity with the current data. Additionally, only self-report measures of sleep disturbance were examined as none of the studies reported objective measures such as polysomnography data. Lastly, the agreement level of the cut-points used for the disparate measures of sleep disturbance has not been assessed. This could be examined in the future for studies that collect a multitude of these measures concurrently to better validate our sleep disturbance measurement harmonization crosswalk for persons with TBI. In future research, and as additional studies are added to FITBIR, it will be important to examine the impact of other factors such as the type of sleep disturbance, presence of a sleep disorder diagnosis, time since injury, mechanism of injury, and other pre-existing or comorbid health conditions on post-TBI sleep outcomes.

Since the start of this project, the number of FITBIR studies with shared data has doubled. As additional studies are added, we expect to be able to make more nuanced investigations. Our team is currently extending the meta-dataset described in this study to include newly shared studies in the FITBIR database. It is our belief that the creation of a widely available repository of TBI data can considerably advance our understanding of the TBI trajectory and the role sleep health plays in recovery, enabling more efficient and comprehensive pooled analyses using individual-participant-level data meta-analytic methods on large datasets [77]. Furthermore, given the heterogeneity in the research on TBI-related care, study-level-data-harmonization efforts focusing on TBI trials could contribute to the development of updated clinical practice guidelines and tailored care models for specific populations and regions. Moreover, we are committed to upholding FAIR data principles, by sharing our harmonization process, making FITBIR summary data easily accessible through interactive components on the FITBIR website (https://fitbir.nih.gov/meta_study_profile/223) (accessed on 21 September 2021), and encouraging other researchers, not just our team, to use data repositories to answer questions about TBI-related outcomes by both contributing original, human-subjects datasets to FITBIR and accessing the publicly available meta-datasets for use in new research endeavors.

## 5. Conclusions

This study demonstrated a method for pooling individual-participant-level data from clinical studies of patients with TBI stored in the publicly accessible FITBIR database to examine the prevalence and risk factors for sleep disturbance in participants with and without mild TBI. Our findings add to mounting evidence that a history of TBI, even if only of mild severity, is independently associated with a greater long-term risk of sleep disturbance. This has significance for clinical care including screening strategies and evaluations for potential diagnoses and treatments of sleep disorders that may have deleterious down-stream effects on other TBI outcomes. As building blocks for future FAIR data and TBI evidence-synthesis efforts, our methods for harmonizing sleep measures across studies and the meta-datasets created in this study are publicly available and can be utilized for future investigations of pooled data analyses on sleep disorders following TBI.

## Figures and Tables

**Table 1:** FITBIR studies and measures in the sleep disorder harmonized cross-sectional dataset.

FITBIR Shared Studies and Measures—Sleep Disorder Outcome							
Study ID	Title	Sample Size	TBI Severity	Sleep Measure	Sleep Variable	Cut Point for Dichotomous Sleep Variable	Included in Analysis
246 [42]	Transforming Research and Clinical Knowledge in Traumatic Brain Injury (TRACK-TBI) Pilot	599	mild TBI, moderate/severe TBI	Rivermead Post-Concussion Symptoms Questionnaire (RPQ)	RPQSleepDistScale	No = 0–2 Yes = 3–4	No
246 [42]	Transforming Research and Clinical Knowledge in Traumatic Brain Injury (TRACK-TBI) Pilot	599	mild TBI, moderate/severe TBI	Posttraumatic Stress Disorder Checklist Civilian Version (PCLC_Standard)	PCLSFallStayAsleepInd	No = 1 Yes = 2–5	No
246 [42]	Transforming Research and Clinical Knowledge in Traumatic Brain Injury (TRACK-TBI) Pilot	599	mild TBI, moderate/severe TBI	TRACK-TBI Neurologic Assessment	NeuroAssmtSlpMoreInd	No = 0 Yes = 1	No
246 [42]	Transforming Research and Clinical Knowledge in Traumatic Brain Injury (TRACK-TBI) Pilot	599	mild TBI, moderate/severe TBI	TRACK-TBI Neurologic Assessment	NeuroAssmtSlpTrblFallInd	No = 0 Yes = 1	No
248 [43]	Progesterone for the Treatment of Traumatic Brain Injury (ProTECT III	882	moderate/severe TBI	Patient Health Questionnaire 8 Item (PHQ 8)	PHQ9SleepImpairScore	No = 0 Yes = 1–3	No
254 [45]	Predictors of PTSD and Post-Concussive Syndrome of OIF/OEF Veterans	80	mild TBI, no TBI	Pittsburgh Sleep Quality Index (PSQI)	PSQITotalScore	No = 0–7 Yes = 8–18	No
263 [47]	CENC Study 1: Observational Study on Late Neurologic Effects of OEF/OIF/OND Combat	1539	mild TBI, no TBI	Pittsburgh Sleep Quality Index (PSQI)	PSQITotalScore	No = 0–7 Yes = 8–18	Yes
264 [48]	CENC Study 25: Assessment of Long-Term Outcome and Disability in Active-Duty Military Prospectively Examined Following Concussive TBI	94	mild TBI, no TBI	Insomnia Severity Index (ISI)	ISITotalScore	No = 0–9 Yes = 10–28	Yes
326 [57]	Automated Comprehensive Evaluation of Mild Traumatic Brain Injury Visual Dysfunction	120	mild TBI, no TBI	Sport Concussion Assessment Tool 3 (SCAT3)	Scat3TroublFallAsleep	No = 0 Yes = 1–6	Yes

**Table 2:** TBI severity, sleep disturbance status, and demographic characteristics of participants from 6 studies [42,43,45,47,48,57].

TBI Category				
	Overall N = 3314 ^1^	No TBI N = 491 ^1^	Mild TBI N = 1920 ^1^	Moderate/Severe TBI N = 903 ^1^
Sleep disorder				
No	1195 (45%)	294 (62%)	640 (39%)	261 (48%)
Yes	1476 (55%)	181 (38%)	1007 (61%)	288 (52%)
Missing	643	16	273	354
Gender				
Male	2674 (81%)	396 (81%)	1612 (84%)	666 (74%)
Female	640 (19%)	95 (19%)	308 (16%)	237 (26%)
Age category				
<25	480 (15%)	69 (14%)	173 (9.0%)	238 (26%)
25–39	1387 (42%)	268 (55%)	851 (44%)	268 (30%)
40–49	688 (21%)	93 (19%)	458 (24%)	137 (15%)
50–64	574 (17%)	54 (11%)	346 (18%)	174 (19%)
65+	180 (5.4%)	5 (1.0%)	89 (4.6%)	86 (9.5%)
Missing	5	2	3	0
Race/ethnicity				
White	1894 (63%)	163 (53%)	1155 (65%)	576 (65%)
Other	1094 (37%)	144 (47%)	635 (35%)	315 (35%)
Missing	326	184	130	12
Population type				
Veteran/Military	1833 (55%)	491 (100%)	1342 (70%)	0 (0%)
Civilian	1481 (45%)	0 (0%)	578 (30%)	903 (100%)

Note ^1^ N (%). TBI = Traumatic Brain Injury.

**Table 3:** TBI severity, sleep disturbance status, and demographic characteristics of participants from three studies included in comparative analyses [47,48,57].

	**TBI Category**		
	**Overall, N = 1753 ^1^**	**No TBI, N = 418 ^1^**	**Mild TBI, N = 1335 ^1^**
Sleep disturbance			
No	724 (42%)	239 (58%)	485 (37%)
Yes	1001 (58%)	172 (42%)	829 (63%)
Missing	28	7	21
Gender			
Male	1527 (87%)	334 (80%)	1193 (89%)
Female	226 (13%)	84 (20%)	142 (11%)
Age category			
<25	103 (5.9%)	63 (15%)	40 (3.0%)
25–39	928 (53%)	217 (52%)	711 (53%)
40–49	444 (25%)	81 (19%)	363 (27%)
50–64	259 (15%)	50 (12%)	209 (16%)
65+	14 (0.8%)	5 (1.2%)	9 (0.7%)
Missing	5	2	3
Race/ethnicity			
Non-Hispanic White	929 (61%)	163 (53%)	766 (63%)
Other	595 (39%)	144 (47%)	451 (37%)
Missing	229	111	118
Population type			
Veteran/Military	1753 (100%)	418 (100%)	1335 (100%)

Note ^1^ N (%). TBI = Traumatic Brain Injury.

**Table 4:** Logistic regression models of three studies included in sleep disturbance comparative analyses [47,48,57].

	OR	CI	*p* Value
Study 263			
Male	0.76	(0.54, 1.07)	0.11
Non-Hispanic White	0.50	(0.40, 0.63)	<0.0001
TBI	1.98	(1.50, 2.62)	<0.0001
Age	0.99	(0.98, 0.99)	0.02
Study 264			
Male	0.76	(0.10, 5.58)	0.78
Non-Hispanic White	1.90	(0.53, 6.81)	0.32
TBI	3.71	(1.24, 11.06)	0.02
Age	1.07	(0.99, 1.15)	0.08
Study 326			
Male	0.54	(0.17, 1.72)	0.30
Non-Hispanic White			
TBI	2.72	(1.06, 6.98)	0.04
Age	1.06	(0.96, 1.17)	0.28
Combined			
Male	0.77	(0.55, 1.07)	0.12
Non-Hispanic White	0.52	(0.42, 0.65)	<0.0001
TBI	2.03	(1.55, 2.65)	<0.0001
Age	0.988	(0.98, 0.99)	0.04
263 vs. 264	0.99	(0.61, 1.61)	0.98

TBI = Traumatic Brain Injury, OR = Odds Ratio, CI = Confidence Interval.

## Data Availability

The meta-data and code from this study are available at https://fitbir.nih.gov/ (accessed on 21 September 2021), by navigating to the Meta Study tab, selecting study “FITBIR: Accelerating Synthesis of TBI Research Using Novel Methods”, and clicking on the Data tab. The meta-data and code are only available to those who have created a FITBIR account. The interactive data visualizations were created using R Shiny Apps and are available to the public on the FITBIR website, https://fitbir.nih.gov/meta_study_profile/223 (accessed on 21 September 2021), by navigating to the Data tab.

## Supplementary Materials

The following supporting information can be downloaded at: https://www.mdpi.com/article/10.3390/brainsci14090921/s1, Figure S1: Sleep Disturbance Outcome by TBI history/severity in FITBIR; total across 6 included studies (N = 3314).

## References

[1] Grima N., Ponsford J., Rajaratnam S.M., Mansfield D., Pase M.P. (2016). Sleep Disturbances in Traumatic Brain Injury: A Meta-Analysis. J. Clin. Sleep Med..

[2] Zuzuárregui J.R.P., Bickart K., Kutscher S.J. (2018). A Review of Sleep Disturbances Following Traumatic Brain Injury. Sleep Sci. Pract..

[3] Nakase-Richardson R., Schwartz D.J., Drasher-Phillips L., Ketchum J.M., Calero K., Dahdah M.N., Monden K.R., Bell K., Magalang U., Hoffman J.M. (2020). Comparative Effectiveness of Sleep Apnea Screening Instruments During Inpatient Rehabilitation Following Moderate to Severe TBI. Arch. Phys. Med. Rehabil..

[4] Montgomery M.C., Baylan S., Gardani M. (2022). Prevalence of Insomnia and Insomnia Symptoms Following Mild-Traumatic Brain Injury: A Systematic Review and Meta-Analysis. Sleep Med. Rev..

[5] Noyes E.T., Tang X., Sander A.M., Silva M.A., Walker W.C., Finn J.A., Cooper D.B., Nakase-Richardson R. (2021). Relationship of Medical Comorbidities to Psychological Health at 2 and 5 Years Following Traumatic Brain Injury (TBI). Rehabil. Psychol..

[6] Hammond F.M., Corrigan J.D., Ketchum J.M., Malec J.F., Dams-O’Connor K., Hart T., Novack T.A., Bogner J., Dahdah M.N., Whiteneck G.G. (2019). Prevalence of Medical and Psychiatric Comorbidities Following Traumatic Brain Injury. J. Head Trauma Rehabil..

[7] Leng Y., Byers A.L., Barnes D.E., Peltz C.B., Li Y., Yaffe K. (2021). Traumatic Brain Injury and Incidence Risk of Sleep Disorders in Nearly 200,000 US Veterans. Neurology.

[8] Wickwire E.M., Schnyer D.M., Germain A., Williams S.G., Lettieri C.J., McKeon A.B., Scharf S.M., Stocker R., Albrecht J., Badjatia N. (2018). Sleep, Sleep Disorders, and Circadian Health Following Mild Traumatic Brain Injury in Adults: Review and Research Agenda. J. Neurotrauma.

[9] Christensen J., Yamakawa G.R., Shultz S.R., Mychasiuk R. (2021). Is the Glymphatic System the Missing Link between Sleep Impairments and Neurological Disorders? Examining the Implications and Uncertainties. Prog. Neurobiol..

[10] Herrero Babiloni A., Baril A.A., Charlebois-Plante C., Jodoin M., Sanchez E., De Baets L., Arbour C., Lavigne G.J., Gosselin N., De Beaumont L. (2023). The Putative Role of Neuroinflammation in the Interaction between Traumatic Brain Injuries, Sleep, Pain and Other Neuropsychiatric Outcomes: A State-of-the-Art Review. J. Clin. Med..

[11] Lowe A., Neligan A., Greenwood R. (2020). Sleep Disturbance and Recovery during Rehabilitation after Traumatic Brain Injury: A Systematic Review. Disabil. Rehabil..

[12] Duclos C., Beauregard M.P., Bottari C., Ouellet M.C., Gosselin N. (2015). The Impact of Poor Sleep on Cognition and Activities of Daily Living after Traumatic Brain Injury: A Review. Aust. Occup. Ther. J..

[13] Holcomb E.M., Towns S., Kamper J.E., Barnett S.D., Sherer M., Evans C., Nakase-Richardson R. (2016). The Relationship Between Sleep-Wake Cycle Disturbance and Trajectory of Cognitive Recovery During Acute Traumatic Brain Injury. J. Head Trauma Rehabil..

[14] Wilde M.C., Castriotta R.J., Lai J.M., Atanasov S., Masel B.E., Kuna S.T. (2007). Cognitive Impairment in Patients with Traumatic Brain Injury and Obstructive Sleep Apnea. Arch. Phys. Med. Rehabil..

[15] Nakase-Richardson R., Sherer M., Barnett S.D., Yablon S.A., Evans C.C., Kretzmer T., Schwartz D.J., Modarres M. (2013). Prospective Evaluation of the Nature, Course, and Impact of Acute Sleep Abnormality after Traumatic Brain Injury. Arch. Phys. Med. Rehabil..

[16] Sandsmark D.K., Kumar M.A., Woodward C.S., Schmitt S.E., Park S., Lim M.M. (2016). Sleep Features on Continuous Electroencephalography Predict Rehabilitation Outcomes After Severe Traumatic Brain Injury. J. Head Trauma Rehabil..

[17] Silva M.A., Nakase-Richardson R., Sherer M., Barnett S.D., Evans C.C., Yablon S.A. (2012). Posttraumatic Confusion Predicts Patient Cooperation during Traumatic Brain Injury Rehabilitation. Am. J. Phys. Med. Rehabil..

[18] Ponsford J., Schönberger M., Rajaratnam S.M. (2015). A Model of Fatigue Following Traumatic Brain Injury. J. Head Trauma Rehabil..

[19] Wickwire E.M., Albrecht J.S., Griffin N.R., Schnyer D.M., Yue J.K., Markowitz A.J., Okonkwo D.O., Valadka A.B., Badjatia N., Manley G.T. (2019). Sleep Disturbances Precede Depressive Symptomatology Following Traumatic Brain Injury. Curr. Neurobiol..

[20] Martindale S.L., Konst M.J., Bateman J.R., Arena A., Rowland J.A. (2020). The Role of PTSD and TBI in Post-Deployment Sleep Outcomes. Mil. Psychol..

[21] Sullan M.J., Crocker L.D., Thomas K.R., Orff H.J., Davey D.K., Jurick S.M., Twamley E.W., Norman S.B., Schiehser D.M., Aupperle R. (2021). Baseline Sleep Quality Moderates Symptom Improvement in Veterans with Comorbid PTSD and TBI Receiving Trauma-Focused Treatment. Behav. Res. Ther..

[22] Bomyea J., Lang A.J., Delano-Wood L., Jak A., Hanson K.L., Sorg S., Clark A.L., Schiehser D.M. (2016). Neuropsychiatric Predictors of Post-Injury Headache After Mild-Moderate Traumatic Brain Injury in Veterans. Headache.

[23] Pattinson C.L., Brickell T.A., Bailie J., Hungerford L., Lippa S.M., French L.M., Lange R.T. (2021). Sleep Disturbances Following Traumatic Brain Injury Are Associated with Poor Neurobehavioral Outcomes in US Military Service Members and Veterans. J. Clin. Sleep Med..

[24] Wei L., Wen Y.T., Thompson H.J., Liu C.Y., Su Y.K., Chen P.Y., Chen C.Y., Chuang Y.H., Lin Y.J., Chen C.T. (2020). Sleep Disturbances Following Traumatic Brain Injury in Older Adults: A Comparison Study. J. Head Trauma Rehabil..

[25] Sullivan K.A., Edmed S.L., Allan A.C., Karlsson L.J., Smith S.S. (2015). Characterizing Self-Reported Sleep Disturbance after Mild Traumatic Brain Injury. J. Neurotrauma.

[26] Albrecht J.S., Wickwire E.M. (2020). Sleep Disturbances among Older Adults Following Traumatic Brain Injury. Int. Rev. Psychiatry.

[27] Rauchman S.H., Zubair A., Jacob B., Rauchman D., Pinkhasov A., Placantonakis D.G., Reiss A.B. (2023). Traumatic Brain Injury: Mechanisms, Manifestations, and Visual Sequelae. Front. Neurosci..

[28] Balba N.M., Elliott J.E., Weymann K.B., Opel R.A., Duke J.W., Oken B.S., Morasco B.J., Heinricher M.M., Lim M.M. (2018). Increased Sleep Disturbances and Pain in Veterans with Comorbid Traumatic Brain Injury and Posttraumatic Stress Disorder. J. Clin. Sleep Med..

[29] Wilkinson M.D., Dumontier M., Aalbersberg I.J., Appleton G., Axton M., Baak A., Blomberg N., Boiten J.-W., da Silva Santos L.B., Bourne P.E. (2016). The FAIR Guiding Principles for Scientific Data Management and Stewardship. Sci. Data.

[30] Stewart L.A., Clarke M., Rovers M., Riley R.D., Simmonds M., Stewart G., Tierney J.F. (2015). Preferred Reporting Items for Systematic Review and Meta-Analyses of Individual Participant Data: The PRISMA-IPD Statement. JAMA.

[31] Dietch J.R., Furst A.J. (2020). Perspective: Cognitive Behavioral Therapy for Insomnia Is a Promising Intervention for Mild Traumatic Brain Injury. Front. Neurol..

[32] Martin A.M., Pinto S.M., Tang X., Hoffman J.M., Wittine L., Walker W.C., Schwartz D.J., Kane G., Takagishi S.C., Nakase-Richardson R. (2023). Associations between Early Sleep-Disordered Breathing Following Moderate-to-Severe Traumatic Brain Injury and Long-Term Chronic Pain Status: A Traumatic Brain Injury Model Systems Study. J. Clin. Sleep Med..

[33] Mysliwiec V., Martin J.L., Ulmer C.S., Chowdhuri S., Brock M.S., Spevak C., Sall J. (2020). The Management of Chronic Insomnia Disorder and Obstructive Sleep Apnea: Synopsis of the 2019 U.S. Department of Veterans Affairs and U.S. Department of Defense Clinical Practice Guidelines. Ann. Intern. Med..

[34] Silva M.A., Calvo D., Brennan E.M., Reljic T., Drasher-Phillips L., Schwartz D.J., Kumar A., Cotner B.A., Taylor D.J., Nakase-Richardson R. (2020). Incidence and Predictors of Adherence to Sleep Apnea Treatment in Rehabilitation Inpatients with Acquired Brain Injury. Sleep Med..

[35] Lequerica A.H., Watson E., Dijkers M.P., Goldin Y., Hoffman J.M., Niemeier J.P., Silva M.A., Rabinowitz A., Chiaravalloti N.D. (2022). The Utility of the Patient Health Questionnaire (PHQ-9) Sleep Disturbance Item as a Screener for Insomnia in Individuals with Moderate to Severe Traumatic Brain Injury. J. Head Trauma Rehabil..

[36] O’Neil M.E. FITBIR: Accelerating Synthesis of TBI Research Using Novel Methods. https://fitbir.nih.gov/meta_study_profile/223.

[37] Chan L. Effect of Aerobic Exercise Training on Cardiorespiratory Function in Patients with TBI (CNRM). https://fitbir.nih.gov/study_profile/205.

[38] Broglio S.P. Concussion Assessment, Research and Education (CARE) Consortium. https://fitbir.nih.gov/study_profile/310.

[39] Rivara F.P., Koepsell T.D., Wang J., Temkin N., Dorsch A., Vavilala M.S., Durbin D., Jaffe K.M. (2011). Disability 3, 12, and 24 Months After Traumatic Brain Injury Among Children and Adolescents. Pediatrics.

[40] Zafonte R., Friedewald W.T., Lee S.M., Levin B., Diaz-Arrastia R., Ansel B., Eisenberg H., Timmons S.D., Temkin N., Novack T. (2009). The Citicoline Brain Injury Treatment (COBRIT) Trial: Design and Methods. J. Neurotrauma.

[41] Lathan C. An Independent, Prospective, Head to Head Study of the Reliability and Validity of Neurocognitive Test Batteries for the Assessment of Mild Traumatic Brain Injury. https://fitbir.nih.gov/study_profile/244.

[42] Yue J.K., Phelps R.R.L., Winkler E.A., Deng H., Upadhyayula P.S., Vassar M.J., Madhok D.Y., Schnyer D.M., Puccio A.M., Lingsma H.F. (2020). Substance Use on Admission Toxicology Screen Is Associated with Peri-Injury Factors and Six-Month Outcome after Traumatic Brain Injury: A TRACK-TBI Pilot Study. J. Clin. Neurosci..

[43] Wright D.W., Yeatts S.D., Silbergleit R., Palesch Y.Y., Hertzberg V.S., Frankel M., Goldstein F.C., Caveney A.F., Howlett-Smith H., Bengelink E.M. (2014). Very Early Administration of Progesterone for Acute Traumatic Brain Injury. N. Engl. J. Med..

[44] Robertson C. Effects of Erythropoietin on Cerebral Vascular Dysfunction and Anemia in Traumatic Brain Injury. https://ctv.veeva.com/study/effects-of-erythropoietin-on-cerebral-vascular-dysfunction-and-anemia-in-traumatic-brain-injury.

[45] Roy M.J., Costanzo M., Gill J., Leaman S., Law W., Ndiongue R., Taylor P., Kim H.S., Bieler G.S., Garge N. (2015). Predictors of Neurocognitive Syndromes in Combat Veterans. Cureus.

[46] Harrison-Felix C. Integrating Traumatic Brain Injury Model Systems Data into the Federal Interagency Traumatic Brain Injury Research Informatics System. https://fitbir.nih.gov/study_profile/255.

[47] Walker W.C., Carne W., Franke L.M., Nolen T., Dikmen S.D., Cifu D.X., Wilson K., Belanger H.G., Williams R. (2016). The Chronic Effects of Neurotrauma Consortium (CENC) Multi-Centre Observational Study: Description of Study and Characteristics of Early Participants. Brain Inj..

[48] Mac Donald C.L., Barber J., Patterson J., Johnson A.M., Dikmen S., Fann J.R., Temkin N. (2019). Association Between 5-Year Clinical Outcome in Patients with Nonmedically Evacuated Mild Blast Traumatic Brain Injury and Clinical Measures Collected within 7 Days Postinjury in Combat. JAMA Netw. Open.

[49] McKenzie L. Evaluation of Spot Light: A Concussion Injury Management App for Youth. https://ctv.veeva.com/study/evaluation-of-spot-light-a-concussion-injury-management-app-for-youth-sports.

[50] Damiano D. Effects of Rapid-Resisted Exercise on Ambulatory Adults with Traumatic Brain Injury (CNRM). https://fitbir.nih.gov/study_profile/271.

[51] Gill J. Biomarkers-Driven Development of Experiemental Therapeutics for Traumatic Brain Injury (CNRM). https://fitbir.nih.gov/study_profile/272.

[52] Gullapalli R. Traumatic Brain Injury Data for FITBIR Informatics System: Maryland Retrospective Dataset. https://fitbir.nih.gov/study_profile/313.

[53] Gullapalli R. Traumatic Brain Injury Data for FITBIR Informatics System: Maryland MagNeTS Prospective Dataset. https://fitbir.nih.gov/study_profile/314.

[54] Sours C. Traumatic Brain Injury Data for FITBIR Informatics System: Maryland MAGNETS Dataset-FMRI TBI Subset. https://fitbir.nih.gov/study_profile/315.

[55] McKee A. Tauopathy Consensus Study of Pathology Images. https://fitbir.nih.gov/study_profile/319.

[56] Okonkwo D.O. Targeted Evaluation, Action, and Monitoring of Traumatic Brain Injury (TEAM-TBI). https://fitbir.nih.gov/study_profile/321.

[57] Capo-Aponte J.E. Automated Comprehensive Evaluation of Mild Traumatic Brain Injury Visual Dysfunction. https://fitbir.nih.gov/study_profile/326.

[58] Weaver L. Brain Injury and Mechanisms of Action of Hyperbaric Oxygen for Persistent Post-Concussive Symptoms after Mild Traumatic Brain Injury (BIMA). https://fitbir.nih.gov/study_profile/363.

[59] Weaver L. Development of Normative Datasets for Assessments Used in Patients with Post Concussive Symptoms Due to Mild Traumatic Brain Injury (NORMAL). https://fitbir.nih.gov/study_profile/364.

[60] Stewart L.A., Tierney J.F. (2002). To IPD or Not to IPD? Advantages and Disadvantages of Systematic Reviews Using Individual Patient Data. Eval. Health Prof..

[61] Cooper H., Patall E.A. (2009). The Relative Benefits of Meta-Analysis Conducted with Individual Participant Data versus Aggregated Data. Psychol. Methods.

[62] Simmonds M., Stewart G., Stewart L. (2015). A Decade of Individual Participant Data Meta-Analyses: A Review of Current Practice. Contemp. Clin. Trials.

[63] Tierney J.F., Stewart L.A., Clarke M., Higgins J.P.T., Thomas J., Chandler J., Cumpston M., Li T., Page M.J., Welch V.A., Cochrane Individual Participant Data Meta-analysis Methods Group (2022). Chapter 26: Individual Participant Data. Cochrane Handbook for Systematic Reviews of Interventions Version 6.3 (Updated February 2022).

[64] Morin C.M. (1993). Insomnia: Psychological Assessment and Management.

[65] Bastien C.H., Vallières A., Morin C.M. (2001). Validation of the Insomnia Severity Index as an Outcome Measure for Insomnia Research. Sleep Med..

[66] Kaufmann C.N., Orff H.J., Moore R.C., Delano-Wood L., Depp C.A., Schiehser D.M. (2019). Psychometric Characteristics of the Insomnia Severity Index in Veterans with History of Traumatic Brain Injury. Behav. Sleep Med..

[67] Simon G.E., Coleman K.J., Rossom R.C., Beck A., Oliver M., Johnson E., Whiteside U., Operskalski B., Penfold R.B., Shortreed S.M. (2016). Risk of Suicide Attempt and Suicide Death Following Completion of the Patient Health Questionnaire Depression Module in Community Practice. J. Clin. Psychiatry.

[68] Simon G.E., Rutter C.M., Peterson D., Oliver M., Whiteside U., Operskalski B., Ludman E.J. (2013). Does Response on the PHQ-9 Depression Questionnaire Predict Subsequent Suicide Attempt or Suicide Death?. Psychiatr. Serv..

[69] Wu Y., Levis B., Riehm K.E., Saadat N., Levis A.W., Azar M., Rice D.B., Boruff J., Cuijpers P., Gilbody S. (2020). Equivalency of the Diagnostic Accuracy of the PHQ-8 and PHQ-9: A Systematic Review and Individual Participant Data Meta-Analysis. Psychol. Med..

[70] Weathers F., Litz B., Huska J., Keane T. (1994). PTSD Checklist-Specific Version. National Center for PTSD. https://www.ptsd.va.gov/professional/assessment/adult-sr/ptsd-checklist.asp.

[71] Weathers F.W. The PTSD Checklist: Reliability, Validity and Diagnostic Utility. Proceedings of the Annual Meeting of the International Society for Traumatic Stress Studies.

[72] Buysse D.J., Reynolds C.F., Monk T.H., Berman S.R., Kupfer D.J. (1989). The Pittsburgh Sleep Quality Index: A New Instrument for Psychiatric Practice and Research. Psychiatry Res..

[73] Fictenberg N.L., Putnam S.H., Mann N.R., Zafonte R.D., Millard A.E. (2001). Insomnia Screening in Postacute Traumatic Brain Injury: Utility and Validity of the Pittsburgh Sleep Quality Index. Am. J. Phys. Med. Rehabil..

[74] Matsangas P., Mysliwiec V. (2018). The Utility of the Pittsburgh Sleep Quality Index in US Military Personnel. Mil. Psychol..

[75] Guskiewicz K.M., Register-Mihalik J., McCrory P., McCrea M., Johnston K., Makdissi M., Dvorák J., Davis G., Meeuwisse W. (2013). Evidence-Based Approach to Revising the SCAT2: Introducing the SCAT3. Br. J. Sports Med..

[76] King N.S., Crawford S., Wenden F.J., Moss N.E., Wade D.T. (1995). The Rivermead Post Concussion Symptoms Questionnaire: A Measure of Symptoms Commonly Experienced after Head Injury and Its Reliability. J. Neurol..

[77] Riley R.D., Lambert P.C., Abo-Zaid G. (2010). Meta-Analysis of Individual Participant Data: Rationale, Conduct, and Reporting. BMJ.

[78] Ventresca M., Schünemann H.J., Macbeth F., Clarke M., Thabane L., Griffiths G., Noble S., Garcia D., Marcucci M., Iorio A. (2020). Obtaining and Managing Data Sets for Individual Participant Data Meta-Analysis: Scoping Review and Practical Guide. BMC Med. Res. Methodol..

[79] VanderWeele T.J., Ding P. (2017). Sensitivity Analysis in Observational Research: Introducing the E-Value. Ann. Intern. Med..

[80] Ahn S., Lobo J.M., Logan J.G., Kang H., Kwon Y., Sohn M.W. (2021). A Scoping Review of Racial/Ethnic Disparities in Sleep. Sleep Med..

[81] Rao V., Bergey A., Hill H., Efron D., McCann U. (2011). Sleep Disturbance after Mild Traumatic Brain Injury: Indicator of Injury?. J. Neuropsychiatry Clin. Neurosci..

[82] Fogelberg D.J., Hoffman J.M., Dikmen S., Temkin N.R., Bell K.R. (2012). Association of Sleep and Co-Occurring Psychological Conditions at 1 Year after Traumatic Brain Injury. Arch. Phys. Med. Rehabil..

[83] Foster S.N., Hansen S.L., Capener D.C., Matsangas P., Mysliwiec V. (2017). Gender Differences in Sleep Disorders in the US Military. Sleep Health.

