# Supplementary material for: Increased Risk for Clinically Significant Sleep Disturbances in Mild Traumatic Brain Injury: An Approach to Leveraging the Federal Interagency Traumatic Brain Injury Research Database
Source: Brain Sci. 2024 Sep 14;14(9):921. doi: 10.3390/brainsci14090921 (PMC11430117; doi:10.3390/brainsci14090921)
Supplement: Supplementary file 1 [file brainsci-14-00921-s001.zip › brainsci-3134969-supplementary.pdf]

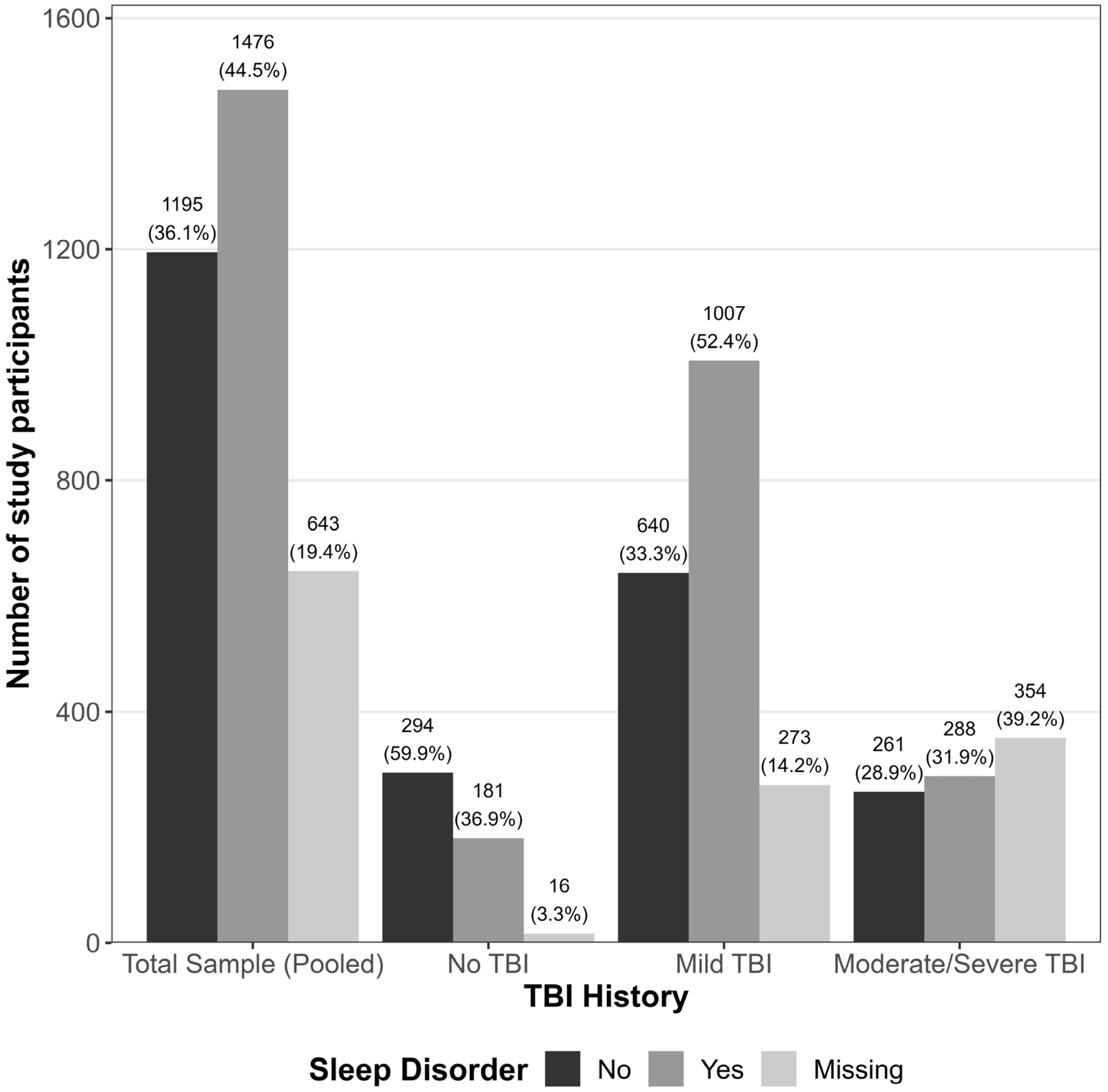

Figure S1. Sleep Disturbance Outcome by TBI history/severity in FITBIR; total across 6 included studies (N = 3314).
